# The Role of Vitamin D in Blood Pressure, Endothelial and Renal Function in Postmenopausal Women

**DOI:** 10.3390/nu5072590

**Published:** 2013-07-09

**Authors:** Zhao-Min Liu, Jean Woo, Sheng-Hui Wu, Suzanne C. Ho

**Affiliations:** 1 Department of Medicine & Therapeutics, the Chinese University of Hong Kong, Hong Kong SAR, 999077, China; E-Mail: liuzhaomin@cuhk.edu.hk; 2 Division of Epidemiology, Department of Medicine, Vanderbilt University Medical Center, Nashville, TN 37203-1738, USA; E-Mail: shenghuiwu@hotmail.com; 3 Division of Epidemiology, The Jockey Club School of Public Health and Primary Care, the Chinese University of Hong Kong, Hong Kong SAR, 999077, China

**Keywords:** vitamin D, blood pressure, endothelial function, renal function

## Abstract

*Background*: Vitamin D is a pro-hormone that plays an essential role in the vasculature and in kidney function. *Aims*: To review the extra-skeletal effects of vitamin D on blood pressure, endothelial and renal function with emphasis on recent findings in postmenopausal women. *Methods*: Included in this review was a PubMed database search for English language articles through March 2013. This review discussed the physiology and definition of vitamin D deficiency, the recent evidence for the role vitamin D in blood pressure, vascular and renal function. *Results*: Experimental and epidemiological data suggest that vitamin D plays an important role in the vasculature and in kidney function. Low vitamin D concentrations appear to significantly associate with hypertension, endothelial and renal dysfunction. However, the results of clinical trials have generally been mixed. Studies specifically conducted among postmenopausal women are limited and findings are still inconsistent. *Conclusions*: Definitive studies are warranted to elucidate the effects of vitamin D supplementation on vascular and renal function and a more detailed work is needed to outline the route, duration and optimal dose of supplementation. It is premature to recommend vitamin D as a therapeutic option in the improvement of vascular and renal function at the current stage.

## 1. Introduction

Vitamin D is a pro-hormone and plays an essential role in a vast number of physiologic processes and clinical consequences [1]. In addition to its traditional effects on calcium homeostasis and bone health, vitamin D receptors (VDR) exist on a very wide range of tissues, including the endothelium, vascular smooth muscle, and cardiomyocytes, suggesting a much wider range of biological functions [1]. However, the association between vitamin D deficiency and non-musculoskeletal conditions, as well as the efficacy of vitamin D supplementation on vascular and renal function, is not adequately characterized. The evidence of causality is inconsistent and inconclusive.

Vitamin D inadequacy is a primary concern for post-menopausal women as they are already predisposed to the osteoporosis and cardiovascular diseases due to decreased oestrogen levels. Menopause represents an important transition in vitamin D requirements due to the dependence of the VDR on oestrogen [2]. In this review, we summarize recent clinical evidence addressing potential mechanisms, epidemiologic associations between vitamin D status and vascular health and renal function, and the effects of supplementation, especially among postmenopausal women.

Studies were identified by searching PubMed for English-language articles through March 2013 by using keywords such as “vitamin D, hypertension, blood pressure, endothelial function, vascular health, chronic kidney disease, renal or kidney function”, alone or in combination. The reference lists of published reports were also searched.

### 1.1. Epidemiology of Vitamin D Status

Vitamin D deficiency is an increasingly recognized public health problem. The direct comparison of data on vitamin D status from various studies are limited by the varying population characteristics, assay methods and the differing thresholds of vitamin D inadequacy. However, all the data suggest that there is a high rate of vitamin D insufficiency. A 2009 study of global vitamin D status reported that serum levels below 30 ng/mL (75 nmol/L) prevailed in every region studied [3]. It has been estimated that 1 billion individuals worldwide are vitamin D insufficient or deficient [4]. Vitamin D insufficiency (serum 25(OH)D level < 30 ng/mL) affects almost 50% of the population worldwide and the prevalence of deficiency (serum 25(OH)D level < 10 ng/mL) ranging from 5% to 25% in the general population [5,6].

The inadequacy of vitamin D affects an even larger proportion of postmenopausal women, particularly those with osteoporosis and a history of fracture [7]. One large international investigation indicated 71% of postmenopausal women with osteoporosis in Eastern Asia had vitamin D inadequacy (25(OH)D < 30 ng/mL). Prevalence rates using this cut-off level were 47% in Thailand, 49% in Malaysia, 90% in Japan, 92% in South Korea and 62.3% in Hong Kong (in adults aged > 50 year) [8]. Very deficient levels (25(OH)D < 10 ng/mL) are most prevalent in South Asia and the Middle East [6,9], possibly due to the wearing of traditional costumes that limit sun exposure, and also extended periods of breastfeeding without vitamin D supplementation. A telephone survey in mid-life Hong Kong Chinese women also showed that 62.3% women actively avoided sunlight exposure by staying indoors or using sunscreen products and parasols [10].

### 1.2. Vitamin D Measurement and Classification

No universal consensus has been reached on which level of serum 25(OH)D reflects optimum vitamin D status. In addition to the large inter-laboratory differences in assays for serum 25(OH)D [11], there are differing recommendations on the serum concentration of 25(OH)D required to maintain the general health [12]. Current International Osteoporosis Foundation guidelines [13] recommended a target level of 30 ng/mL (75 nmol/L), which is associated with maximal suppression of parathyroid hormone (PTH), and defined vitamin D insufficiency as 25 (OH)D levels less than 20 ng/mL (50 nmol/L) and deficiency as levels less than 10 ng/mL (25 nmol/L). However, a report from the Institute of Medicine recommended that a serum 25(OH)D level of 20 ng/mL (50 nmol/L) was sufficient to ensure bone health. The Institute of Medicine report does not support the recommendation that all adults should have levels of 25(OH)D greater than 30 ng/mL (75 nmol/L). The report also notes that higher levels of vitamin D may lead to adverse health outcomes, including kidney stones and renal impairment [14]. Although there is still debate on how to classify vitamin D status, it is generally accepted that serum 25(OH)D levels of 10 and 30 ng/mL are the cut-off values for deficiency and insufficiency, respectively [15].

Several commercial methods are available for serum 25(OH)D measurements. The international Vitamin D Quality Assessment Scheme [16] demonstrated that most commercial 25(OH)D measurements were capable of producing reliable results, but the results were operator-dependent and most methods had notable bias compared with HPLC methods. The variation between laboratories may be as high as 30%. Liquid chromatography tandem mass spectroscopy (LC–MS) is considered the “gold standard” [17]. Other simpler methods, such as radioimmunoassay (RIA), enzyme-linked immunoassay (ELISA), and chemiluminescence assay may not measure all circulating forms of vitamin D.

### 1.3. Vitamin D Metabolism

In humans, skin synthesis of vitamin D from sunlight exposure is the major source of vitamin D (80%–90%) in humans under natural conditions [18]. The dietary supply of vitamin D is minor (10%–20%) compared to skin formation but can become an important source of vitamin D with supplementation [4].

Vitamin D is a collection of fat-soluble steroids. There are two types of physiologically important vitamin D: cholecalciferol (vitamin D_3_) and ergocalciferol (vitamin D_2_). D_2_ is typically obtained from fortified foods and vitamin sources [19], while D_3_ is mainly synthesised in the skin from 7-dehydrocholesterol upon exposure to UVB (290–320 nm) [20]. In the circulation, vitamin D is metabolised to 25-hydroxyvitamin D [25(OH)D] in the liver and further metabolised to its biologically active form, 1,25-dihydroxyvitamin D [1,25(OH)_2_D] in the kidney by 25-hydroxyvitamin D-1α-hydroxylase (CYP27B1).

The concentration of 1,25(OH)_2_D is regulated by a variety of factors including serum PTH, calcium, and phosphate [20,21]. PTH increases the activity of CYP27B1, resulting in increased production of 1,25(OH)_2_D. The higher 1,25(OH)_2_D_3_ level can also inhibit PTH secretion, completing the feedback loop. 25(OH)D is the major circulating form of vitamin D. The majority of circulating 25(OH)D and 1,25(OH)_2_D is bound to vitamin D binding protein (DBP) (80%–90%) and albumin (10%–20%), while a small fraction of both 25(OH)D (0.02%–0.05%) and 1,25(OH)_2_D (0.2%–0.6%) is free [17].

The serum 25(OH)D level is the best indicator of overall vitamin D status since its long half-life (10–27 days following oral or intravenous administration and 1–3 months based on pharmacodynamic response, a more clinically-oriented half-life [22,23]) and its metabolism reflects total vitamin D from dietary intake and sunlight exposure, as well as the conversion of vitamin D from adipose stores in the liver [24,25]. 1,25(OH)_2_D circulates in much lower concentrations than 25(OH)D but has much greater affinity for the vitamin D receptor (VDR) and is biologically more potent. Although 25(OH)D requires additional hydroxylation in the kidney to become its active form, serum concentrations of 1,25(OH)_2_D should never be used to determine vitamin D status. This is because of the short half-life of 1,25(OH)_2_D in the circulation (<4 h), its concentrations are ~1000-fold less than those of 25(OH)D and most importantly, the 1,25(OH)_2_D level would be normal or even elevated as a result of vitamin D deficiency or secondary hyperparathyroidism [4].

The active form of 1,25(OH)2D binds to VDR. VDR is nearly ubiquitously expressed. In addition to its traditional effects on bone health, there is accumulating evidence to suggest that the VDR has a broad spectrum of effects on various cell types including the endothelium [26], vascular smooth muscle [27,28], and cardiomyocytes [29]. Approximately 3% of the human genome is directly or indirectly regulated by the vitamin D endocrine system, which supports the idea that vitamin D insufficiency has widespread adverse consequences for human health [30].

### 1.4. Risk Factors with Vitamin D Deficiency

The principal causes of low vitamin D levels are limited cutaneous synthesis due to inadequate sun exposure (sunscreen use, institutionalized or homebound status) or combined with low dietary intake of vitamin D rich foods [7] including fortified milk [4]. Other risk factors include aging, pigmented skin, smoking, obesity, air pollution, abnormal intestinal function, malabsorption, or reduced synthesis or increased degradation of vitamin D due to chronic liver or renal disease [4,7].

Older people have lower dermal synthesis of vitamin D. Even similar exposure to sunlight, a person aged 70 y produces 75% less vitamin D than a person aged 20 [31]. Persons with a high body mass index are also susceptible to low vitamin D levels because of the decreased bioavailability of vitamin D that is stored in excess adipose tissue. Gender disparities exist with women having lower 25(OH)D levels than men [32]. Vitamin D inadequacy could also be affected by the ethnic and culture factors. Asian women often have a lactase deficiency and lower fortified milk consumption, and often avoid the sun exposure and skin pigmentation by using sunscreening cosmetics and parasols [10], which increase their risk of vitamin D insufficiency. Urbanization is also an important risk factor for an inadequate vitamin D status, which often leads to insufficient outdoor activities [33] and is associated with highly polluted air in some cities [34].

### 1.5. Vitamin D Supplementation Guidelines

Foods naturally containing rich vitamin D are limited. Oily fish (salmon, mackerel, and sardines) and cod liver oil are good sources of vitamin D_3_. Other food source includes egg yolk, fortified milk and orange juice, some cereals, mushrooms and cheese [20,35]. It has been estimated that for every 100 IU of vitamin D ingested, the blood level of 25(OH) D increases by around 1 ng/mL (2.5 nmol/L) [36]. The estimate could be varied with starting level of vitamin D, body composition, dose quantity and frequency, or the supplemental form of vitamin D [37] Data from NHANES-III indicate a “J-shaped” association between 25(OH)D levels and mortality, with slightly increasing mortality in those with supraphysiological 25(OH)D levels. However, other data indicate that particularly high levels of vitamin D are optimal for cancer prevention [38,39].

The US Institute of Medicine concluded that serum 25(OH)D of 20 ng/mL or more will cover the requirements of 97.5% of the population and recommended Dietary Allowance at 600 IU per day for people aged 1–70 year and 800 IU per day for older adults [40]. The US Endocrine Society’s Clinical Practice Guideline suggested that 600–1000 IU per day for children aged one year or more, and 1500–2000 IU per day for adults aged 19 years or more to maintain 25(OH)D above the optimal level of 30 ng/mL [41]. The guideline also recommended screening for vitamin D deficiency in individuals at risk for deficiency and concluded that there was not sufficient evidence to recommend screening individuals who are not at risk for deficiency or to prescribe vitamin D to attain the non-calcaemic benefit for cardiovascular protection [41]. Difference in the recommendations reflects different goals and views on current evidence.

## 2. Vitamin D and Blood Pressure

The effect of vitamin D on blood pressure could be one of the potential mechanisms underlying the link between vitamin D and cardiovascular diseases. The 1α-hydroxylase enzyme that converts 25(OH)D to 1,25(OH)_2_D is expressed in human endothelial and vascular smooth muscle cells which have special relevance in the genesis of hypertension. *In vitro* and animal studies demonstrated that vitamin D appears to have antihypertensive and vasculoprotective effects via multiple pathways [42]. The antihypertensive properties of vitamin D include renoprotective effects, suppression of the rennin-angiotensin-aldosterone system (RAS), direct effects on vascular cells, and effects on calcium metabolism, including prevention of secondary hyperparathyroidism and hypocalcemia [43]. Vitamin D might also act as a negative regulator of the renin gene, and low vitamin D may increase the expression of the RAS [44].

### 2.1. Cross Sectional Data

The association between 25(OH)D levels and arterial hypertension has been assessed in a number of cross-sectional studies [45,46,47,48,49,50,51,52,53]. Most, though not all, of the observational data support the links between low 25-(OH)-D levels and a higher risk of hypertension [54,55].

Several large scale cross-sectional investigations revealed an inverse association of vitamin D levels with prevalence of hypertension. The Third National Health and Nutrition Examination Survey (NHANES-III) [45] among 12,644 non-institutionalized US civilians showed that systolic BP was inversely and significantly correlated with 25(OH)D levels. The mean systolic and diastolic BP reduced 3.0 and 1.6 mm Hg (*p* < 0.05) for participants in the highest quintile compared with the lowest after adjusting for potential cofounders. Subgroup analyses also confirmed that age adjusted systolic BP was significantly lower in individuals with vitamin D sufficiency and the impact of vitamin D deficiency was highly significant in the elderly (age > 50 years) relative to younger individuals [46,47]. The German National Health Interview and Examination Survey [48], 1958 British Birth Cohort [49] and US adolescent population (NHANES 2001 and 2006) [56] all demonstrated that 25(OH) D was inversely associated with the prevalence of hypertension.

A large cohort study [57] in Shanghai, China also reported an association between a lower risk of hypertension and the highest quintile of 25(OH)D (OR = 0.16 for vitamin D ≥ 50.6 nmol/L compared with <23.5 nmol/L; *p* for trend 0.02). However, among women, no significant associations were found for BP parameters and hypertension with 25(OH)D level. Another two observational studies among older populations, the Longitudinal Aging Study Amsterdam [58] and the Ranch Bernardo Study [51] reported the lack of a significant association between vitamin D status and BP. The lack of association might be attributable to the relatively high baseline levels of 25(OH)D among the study participants. Despite the inconsistent findings from some cross-sectional investigations, the majority of studies with large sample sizes have demonstrated an inverse relationship between 25(OH)D levels and BP [43].

### 2.2. Cohort Studies

A few prospective studies [51,59,60,61,62] have addressed the association of 25(OH) D and change in blood pressure or new-onset hypertension. The findings are still inconsistent.

A 5-year prospective study among 1471 older women (mean age 74 year) fail to show a significant association between initial levels of 25(OH)D and change of BP. However, when the analyses were adjusted for vitamin D supplement use, greater initial levels of 25(OH)D (≥20 ng/mL compared to <20 ng/mL) were paradoxically associated with larger increases in BP [59]. In a cohort [60] of 4863 postmenopausal women who were recruited into the Women’s Health Initiative between 1993 and 1998, serum levels of 25(OH)D were also not related to changes in BP, and evidence for an association with lower risk of incident hypertension was weak.

Wang *et al*. [61] studied more than 1700 Framingham Offspring Study participants (mean age 59 years; 55% women; all white) and suggested that low serum vitamin D levels appear to interact with pre-existing hypertension to dramatically raise the risk of future cardiovascular events. In another large cohort [62] including 77,531 women who were followed for 18 years, the multivariable relative risks comparing the lowest to highest deciles were 1.57 (95% CI: 1.44 to 1.72) in women. However, this study was limited since the 25(OH)D levels of study populations were predicted using sun exposure or nutritional vitamin D intake as a surrogate.

### 2.3. Clinical Trials on Vitamin D Supplementation and BP

A number of randomized controlled trials (RCT) evaluated the impact of vitamin D supplementation on BP, however the results are inconclusive. Several large scale trials specifically conducted among postmenopausal women also reported inconsistent findings.

In the largest trial [63] in this field—the Women’s Health initiative (WHI)—36,282 postmenopausal women were randomly assigned to receive either 400 IU vitamin D plus 1000 mg calcium daily or placebo. After seven years’ follow-up, changes in systolic and diastolic BP, as well as the frequency of incident hypertension, were not significantly different between the intervention and placebo groups. However, the findings may not rule out an effect of vitamin D on blood pressure since 80% of patients enrolled in this trial were normotensive at baseline, the dose (400 IU/day) of vitamin D_3_ used was relatively low to sufficiently increase the 25(OH)D concentrations, and adherence to treatment was also low with only 60%–63% in the first three years and 59% at the end of the trial.

Similarly, a one year RCT in UK among 305 healthy postmenopausal women aged 60–70 years also reported that daily doses of vitamin D3 at 400 or 1000 IU/day for one year did not affect conventional markers of cardiovascular disease (CVD) risk including lipids, blood pressure and endothelial markers *etc*. [64]. However, another RCT among 148 elderly women (aged 70 year or above) with vitamin D deficiency (25[OH]D levels < 25 ng/mL) demonstrated a significant antihypertensive effect of vitamin D [65]. In this study, subjects were assigned to receive either 1200 mg of calcium or 1200 mg of calcium plus 800 IU of vitamin D daily. After eight weeks of treatment, a significant reduction in SBP (7.4 mmHg, *p* = 0.02) was observed in the vitamin D plus calcium group, but no significant difference in diastolic BP [65]. Another trial included 34 vitamin-D deficient patients with type 2 diabetes also reported positive findings. Subjects were randomly assigned to receive a single dose of 100,000 IU vitamin D or placebo [66]. After eight weeks of follow-up, the mean office systolic BP was 14 mmHg lower in the vitamin D group (*p* = 0.001).

A meta-analysis including 11 RCT provided little support for a positive effect of vitamin D supplementation on blood pressure [67]. Most of these studies were small of variable methodological quality and indicated a significant heterogeneity. Only a small effect of vitamin D on diastolic BP (3.1 mmHg, 95% CI: 5.5 to 0.6) was found with no significant fall in systolic BP in studies of hypertensive patients [67]. The inconclusive findings could be because the majority of trials included were not primarily or adequately designed to evaluate the antihypertensive effects of vitamin D and participants had 25(OH)D levels within the normal range and were largely free from arterial hypertension at baseline. In addition, BP was generally measured in the office, and more sophisticated BP measurements such as ambulatory blood pressure, were not used.

In summary, although experimental and observational studies favor the hypothesis that vitamin D sufficiency promotes the lowering of arterial BP, findings from clinical trials are still inconclusive, especially among postmenopausal women. In general, the antihypertensive effects of vitamin D seem to be particularly prominent in vitamin-D-deficient patients with elevated blood pressure. Further large and well designed randomized, placebo-controlled trials are needed to clarify the effect of vitamin D on blood pressure.

## 3. Vitamin D and Endothelial Function

Endothelial dysfunction is an early vascular pathology and characterized by a change in the properties of the endothelium toward decreased vasodilatation and the creation of a proinflammatory and prothrombotic state [68]. Endothelial function is a valuable surrogate marker of cardiovascular risk [68]. It plays an important role in the pathogenesis of atherosclerosis contributing to plaque initiation and progression [69], as well as increasing arterial stiffness [70].

There are many potential mechanisms mediating a vitamin D effect on endothelial function. Vitamin D may improve endothelial function indirectly by reducing BP, which may in turn be due to its suppressing renin-angiotensin system [42] and/or to its decreasing vascular resistance [71]. Vascular smooth muscle and endothelial cells express VDR as well as 1α-hydoxylase [28], allowing for autocrine production of 1,25(OH)_2_D, which may act at the local level to modulate the effects of inflammatory cytokines on the vasculature [72], such as decreasing endothelial adhesion molecules, increasing nitric oxide production [73] and reducing platelet aggregation [74,75]. Other potential mechanisms linking vitamin D to vascular health include the decrease in oxidative stress [76], attenuation of NF-κB activation [77] and reduction of PTH [78]. Vitamin D deficiency is also associated with higher circulating concentrations of matrix metalloproteinase-9 which controls vascular wall remodeling [79].

### 3.1. Observational Studies

Most observational studies have shown associations between low circulating levels of vitamin D and endothelial dysfunction. In a large study of 554 healthy individuals, serum vitamin D levels were independently associated with brachial artery flow mediated vasodilatation (FMD) and arterial stiffness after adjusting for age, sex, race, BMI, serum lipid levels, plasma C-reactive protein and medications [80]. A small study involving 23 asymptomatic subjects demonstrated that subjects with significant vitamin D deficiency have impaired brachial artery FMD, which improved after vitamin D replacement therapy [76]. Recently, a step-wise change in FMD according to vitamin D status was demonstrated and an inverse association between serum 25(OH)D levels and vascular inflammatory markers observed [77].

In addition to healthy individuals, vitamin D levels can influence endothelial function in disease states. Impaired brachial artery FMD was documented in 280 type 2 diabetic individuals who had low serum vitamin D concentrations [80]. Another study [81] among 66 obese Caucasian children aged 7–14 years indicated that Vitamin D status is linked to biomarkers of oxidative stress, inflammation, and endothelial activation, among which the obese children with vitamin D insufficiency had substantially elevated malondialdehyde, myeloperoxidase, 3-nitrotyrosine, interleukin-6, and sVCAM-1 levels.

### 3.2. Clinical Trials on Vitamin D and Endothelial Function

Clinical trials using vitamin D as supplement produced inconsistent findings (Table 1) [66,76,82,83,84,85,86,87,88,89,90]. Several RCTs [76,86,87,88,89] have shown vitamin D supplementation improves endothelial function while others not [82,83,84,90]. The discrepancies may be due to various vitamin D dosages or dosing interval, study duration, outcome measures, sample size and participant features *etc*.

**Table 1:** Randomized controlled trials on vitamin D supplementation and endothelial function.

Authors year	Sample size	Participants	duration	Intervention groups	Outcome measures
Schleithoff, S.S. *et al*., 2006 [89]	123	Patients with CHF	9 months	50 μg vitamin D_3_ plus 500 mg Ca/day or placebo plus 500 mg Ca/day	Vitamin D_3_ reduces the inflammatory and endothelial biomarkers.
Sugden, J.A. *et al*., 2008 [66]	34	Patients with Type 2 diabetes and low serum 25(OH) D levels (<50 nmol/L), with average age of 64 year	8 weeks	A single large dose of vitamin D_2_ 100,000 IU or placebo	Vitamin D significantly improved brachial FMD by 2.3% independent of BP change.
Tarcin, O. *et al*., 2009 [76]	23	Vitamin D-deficient subjects (25(OH)D < 25 nmol/L)	3 months	300,000 IU im monthly for 3 months	Vitamin D has favorable effects on FMD.
Dong, Y. *et al*., 2010 [87]	49	Normotensive black boys and girls, mean age 16.3 year	16-week	2000 IU daily vitamin D_3_ supplementation	Significant improvements in PWV with vitamin D_3_ supplementation.
Witham, M.D. *et al*., 2010 [90]	61	Patients with type 2 diabetes and baseline 25(OH) D levels < 100 nmol/L	16 weeks	A single oral dose of placebo or vitamin D_3_ 100,000 IU or 200,000 IU	No significant difference in the primary outcome of endothelial function.
Harris, R.A. *et al*., 2011 [86]	57	African Americans	16 weeks	60,000 IU monthly supplementation of oral vitamin D_3_ and placebo group	Significant improvements on FMD in the vitamin D group (1.8 ± 1.3%).
Shab-Bidar *et al*., 2011 [85]	100	Patients of type 2 diabetes	12 weeks	Vitamin D_3_-fortified yogurt drink (containing 170 mg calcium and 500 IU/250 mL), or 170 mg calcium and no vitamin D/250 mL), twice a day	A significant improvement in endothelin-1, E-selectin and MMP-9 by vitamin D3-fortified yogurt drink.
Witham, M.D. *et al*., 2012 [88]	58	Patients with stroke and baseline 25-(OH) D levels < 75 nmol/L, with mean age 67 year	16 weeks	A single large dose of 100,000 IU oral vitamin D_2_ or placebo	FMD was increased at 8 weeks (6.9% *vs*. 3.7%, but not at 16 weeks after vitamin D supplementation.
Stricker, H. *et al*. 2012 [82]	62	Patients with peripheral arterial disease (PAD) and low 25 (OH) D level (<30 ng/mL)	1 month	A single large dose of 100,000 IU oral vitamin D_2_ or placebo	No effect on endothelial function and arterial stiffness.
Longenecker C.T. *et al*., 2012 [83]	45	HIV-patients with baseline Vitamin D deficient (</=20 ng/mL)	12 weeks	vitamin D_3_ 4000 IU daily or placebo	No effect on FMD.
Gepner, A.D. *et al*., 2012 [84]	114	Post-menopausal women with serum 25(OH) D concentrations 10–60 ng/mL	4 months	Vitamin D_3_ 2500 IU or placebo	No significant effect on FMD, PWV or AIx.

CHF denotes congestive heart failure; BP denotes blood pressure; Ca denotes calcium; FMD denotes flow mediated dilation; PWV denotes pulse wave velocity; AIx denotes augmentation index; im denotes intramuscular injection.

A study by Harris *et* *al*. [86] reported that supplementation with 60,000 IU per month of oral D_3_ for 16 weeks is effective in improving vascular endothelial function in African-American adults with significant improvements in FMD (1.8 ± 1.3%). Another study in 42 subjects with vitamin D insufficiency, normalization of serum 25(OH)D was associated with increases in reactive hyperemia index (0.38 ± 0.14, *p* = 0.009) and a decrease in mean arterial pressure (4.6 ± 2.3 mmHg, *p* = 0.02) after 6 months of supplementation [91]. A 16 week RCT [87] indicated that vitamin D supplementation (2000 IU/day) decreased arterial stiffness by the mean pulse wave velocity reduced from 5.41 m/s at baseline to 5.33 m/s (*p* = 0.031). In another RCT [66], which was conducted among diabetic patients with baseline 25(OH)D insufficiency, a single dose of 100,000 IU vitamin D_2_ significantly improved FMD of the brachial artery by 2.3% at 8 weeks. A 9-month RCT [89] among 123 patients with congestive heart failure also indicated that 50 μg vitamin D_3_ plus 500 mg Ca per day notably improves cytokine profiles and decreases PTH level compared with calcium alone.

In contrast, a 16-week RCT [90] of vitamin D replacement among 61 diabetic subjects, both low- and high-dose vitamin D_3_ supplementation (100,000 and 200,000 IU) failed to modulate FMD, although an effect on BP was noted. A one month pilot study [82] among 62 patients with peripheral arterial disease, a single large dosage of 100,000 IU oral vitamin D_2_ indicated nil effect on endothelial function and arterial stiffness. The non-significant finding of the pilot trial might be due to the short duration or underpowered study participants. Clinical trials specifically conducted among postmenopausal women are limited. One RCT among 114 post-menopausal women with serum low vitamin D status (25(OH)D >10 and <60 ng/mL), reported that 2500 IU Vitamin D_3_, daily for 4 months did not improve endothelial function, arterial stiffness, or inflammation [84]. Multivariable models showed no significant interactions between treatment group and Vitamin D status (<30 ng/mL). Further studies applying different dosages of vitamin D, with adequate sample size and study duration, among postmenopausal women with confirmed vitamin D deficiency are necessary to elaborate the effectiveness of vitamin D on vascular function in women after menopause.

## 4. Vitamin D and Renal Function

The kidney plays an essential role in vitamin D metabolism in circulation [92]. Chronic kidney disease (CKD) is a condition characterized by a gradual loss of kidney function over time. The abnormalities in vitamin D metabolism could contribute to the development of mineral and skeletal disorders, elevations in PTH, hypertension, systemic inflammation, and finally result in renal and cardiovascular damage [93]. The 2009 KDIGO (Kidney Diseases: Improving Global Outcomes) clinical practice guidelines recommended correcting 25(OH)D deficiency and insufficiencies for the general population [94].

The reasons for this marked vitamin D deficiency in CKD are multi-factorial. CKD can induce a progressive loss of the capacity of the kidney not only to convert 25(OH)D to circulating calcitriol (the vitamin D hormone), but also to maintain serum 25(OH)D levels for non-renal calcitriol synthesis. The resulting calcitriol and 25(OH)D deficiency associates directly with accelerated disease progression and death [93,95]. Another interesting hypothesis is that urinary loss of 25(OH)D-VDBP (the main plasma carrier of vitamin D in circulation) associated with proteinuria and reduced megalin-mediated uptake might result in vitamin D deficiency. Alternatively, reduced levels of 25(OH)D might be a result of compromised endogenous pre-vitamin D production in the skin due to severe renal dysfunction [96] or simply lack of outdoor sunlight exposure due to morbidity.

Most [97,98,99], though not all [75], the observational studies have demonstrated 25(OH)D deficiency is independently associated with impaired renal function. A cross-sectional analysis of the NHANES III data [100] revealed an association between vitamin D deficiency and increased risk of albuminuria in the US adult population. The possible explanation could be that vitamin D may have an intrinsic antiproteinuric activity, or the fraction of vitamin D which is bound to albumin is lost during albuminuria. Cohort studies among patients with end stage renal disease (ESRD) also indicated higher 25(OH)D or 1,25(OH)_2_D levels were associated with decreased overall mortality [101,102,103]. Most of the current observational findings are from patients with CKD or severe kidney dysfunction; studies among individuals with mildly or moderately declined renal function especially among postmenopausal women are few.

Results from animal experiments demonstrated reno-protective effects of both active vitamin D and its analogues [104]. The favourable effects are mediated by the vitamin D receptor (VDR) and appear to act by regulating multiple pathways including the renin–angiotensin system (RAS), NF-κB, Wnt/β-catenin and some key structural proteins [105]. Therefore, addition of vitamin D to conventional therapy may present a promising treatment modality that extends its classical role in the maintenance of mineral homeostasis [106,107].

Randomized, controlled trials addressing renoprotective potential of vitamin D are not adequately studied. Indirect evidence suggests that treatment with vitamin D receptor activators confers a considerable survival advantage in hemodialysis patients [108,109,110]. A large scale cohort study [110] among chronic hemodialysis patients demonstrated that patients who received injectable vitamin D had a significant survival advantage (20%) than the non-vitamin D group. The incidence of cardiovascular-related mortality was significantly reduced with vitamin D injection from 14.6/100 to 7.6/100 person-years (*p* < 0.001). Another 16-month trial [108] among Latin America countries also showed that the 7203 patients who received oral active vitamin D had significant reductions in overall, cardiovascular, infectious and neoplastic mortality compared to the 8801 patients that had not received vitamin D. Several trials using vitamin D analog paricalcitol in patients with CKD have noted significant reduction in albuminuria and the effects were independent of hemodynamics or parathyroid hormone suppression [111,112,113]. In a multinational, 24-week RCT [111] among patients with type 2 diabetes and albuminuria, intake of 2 mug paricalcitol showed a safe and sustained reduction in urinary albumin-to-creatinine ratio (UACR) ranging from −18% to −28% (*p* = 0.014 *vs*. placebo). Another pilot trial [113] also showed that ingestion of paricalcitol for 1 month significantly reduces albuminuria and inflammation levels in 24 patients with CKD and the effect was independent of hemodynamics or parathyroid hormone suppression. Large, randomized, controlled clinical trials to assess the renoprotective potential of vitamin D are expected in the near future.

Although no consensus exists on the optimal levels of 25(OH)D measured in the serum, the US Institute of Medicine recommended that mean 25(OH)D levels should be >20 ng/mL in the general population. It might be reasonable to suggest slightly higher levels (up to 30 ng/mL) in patients with CKD [114] for extra renal production of 1,25(OH)_2_D_3_ and regulation of PTH secretion. In CKD, supplementation with vitamin D is recommended at the inception of the disease, with the addition of 25(OH)D beginning in Stage 3 [115]. 

## 5. Vitamin D Intoxication (VDI)

In clinical trials, vitamin D toxicity was not observed with doses of up to 10,000 IU per day [116]. Serum 25(OH)D levels above 150 ng/mL are considered as VDI [117]. There is a wide margin between the level of 25(OH)D required for vitamin D adequacy (>30 ng/mL) and the level of toxicity (>150 ng/mL). The adverse effects with VDI are mainly related to serum hypercalcemia and its duration, which can cause reversible hypertension [4]. When the calcium concentration exceeds 14 mg/dL, emergency intervention is necessary since hypercalcemia will cause organ dysfunction. Since vitamin D is stored in fat tissues, effects of toxicity may last for months despite the removal of the exogenous source of vitamin D.

## 6. Conclusions

The observational data presented in this review suggest an association between low vitamin D status and increased blood pressure, endothelial and renal dysfunction; however, clinical trials have reported inconsistent findings. The discordant findings of RCTs could be due to the differences in features of participants, their adherence to the supplements, study duration, various dosage regimes or dosing interval of vitamin D, the concomitant calcium intake, or the primary outcome that was measured [118]. Moreover, a number of RCTs have paid little attention to the baseline vitamin D status and dose adequacy and studies tested the dose-response relationship were few [119].

Although a single hormone like vitamin D seems unlikely to play a substantial role in preventing or ameliorating the diverse range of diseases [120], future studies from appropriately powered and controlled clinical trials to examine the dose-response effect, selecting patients at risk for vascular and renal dysfunction in the context of initial vitamin D deficiency, and defining the appropriate product regimes and dosing interval, are much needed before vitamin D can be recommended as an auxiliary or therapeutic option for the improvement of vascular and renal function.
